# Orientation-Dependent Displacement Sensor Using an Inner Cladding Fiber Bragg Grating

**DOI:** 10.3390/s16091473

**Published:** 2016-09-11

**Authors:** Tingting Yang, Xueguang Qiao, Qiangzhou Rong, Weijia Bao

**Affiliations:** Physics Department, Northwest University, Xi’an 710069, China; ttyang@stumail.nwu.edu.cn (T.Y.); vj199107@163.com (W.B.)

**Keywords:** inner cladding-FBG inscription, femtosecond laser, orientation bending

## Abstract

An orientation-dependent displacement sensor based on grating inscription over a fiber core and inner cladding has been demonstrated. The device comprises a short piece of multi-cladding fiber sandwiched between two standard single-mode fibers (SMFs). The grating structure is fabricated by a femtosecond laser side-illumination technique. Two well-defined resonances are achieved by the downstream both core and cladding fiber Bragg gratings (FBGs). The cladding resonance presents fiber bending dependence, together with a strong orientation dependence because of asymmetrical distribution of the “cladding” FBG along the fiber cross-section.

## 1. Introduction

Displacement measurement is one of the critical issues in engineering applications (such as industrial and health monitoring) [1,2,3,4]. Fiber Bragg grating, as a smart optical device, has a great performance on monitoring displacement (bending) [5,6,7,8]. For instance, FBG inscription within two eccentric cores of a polymer fiber has been applied to measure fiber bending [9]. Besides, a bend sensor based on FBG inscribed in a single-mode fiber within a depressed-index structure has been proposed and experimentally demonstrated [10]. Recently, Villatoro et al. reported a direction-dependent sensor based on a fiber within an asymmetric three-core [11]. For these sensors, fiber-bending variation is retrieved from the wavelength response. Therefore, the temperature perturbation and complex interrogator are inescapable. A power-referenced interrogation technique is a suggested solution to those problems. Especially, cladding modes that are converted from the core mode with the method of core-mismatch [12,13] and post-processing [14,15] significantly lose with fiber bending. Tilted fiber Bragg grating (TFBG) is another typical device based on the coupling of the core mode to the amount of backward-propagating cladding modes for bending sensing [16,17,18]. In addition, long period gratings (LPGs) have also been widely employed to intrinsically address the coupling of the core-to-cladding mode whose transmission resonant dip is sensitive to bending [19,20,21]. However, when working with cladding modes, the discontinuity technique needs to be provided for ensuring the coupling of core-to-cladding modes, which may enlarge the light signal-to-noise ratio and make sensors fabrication complex. 

Another alternative technique based on FBG inscription over fiber cladding using a femtosecond laser side-illumination technique has been demonstrated and successfully utilized for displacement measurement in our previous work [22]. With the high-intensity and ultrashort pulses of the femtosecond laser, the nonlinear light-material interaction involving nonlinear multiphoton absorption and ionization will be induced, and the grating region can be formed in the fiber cladding [22]. The cladding-FBG resonance shows a great response to bending or deflecting on the fiber due to induced propagation loss and a good orientation dependence because of the asymmetrical distribution of the cladding FBG along the fiber cross-section [23]. Moreover, power fluctuations (originating from the light source, transmission lines, and connectors) can be effectively canceled out by monitoring the bend-insensitive core-mode reflection.

In this paper, we report the performance for a FBG inscription over a fiber core and inner depressed-index cladding in a section of multi-cladding fiber (MCF) via the femtosecond laser side-illumination technique. This construction seems similar to the sensor proposed in the previous report [24]. The device has a simple fabrication and a great spectral quality. The cladding mode-assisted coupling can be used to measure the fiber bending with high sensitivity and definite orientation dependence.

## 2. Fabrication and Principle of QCF-FBG

**Figure 1:**
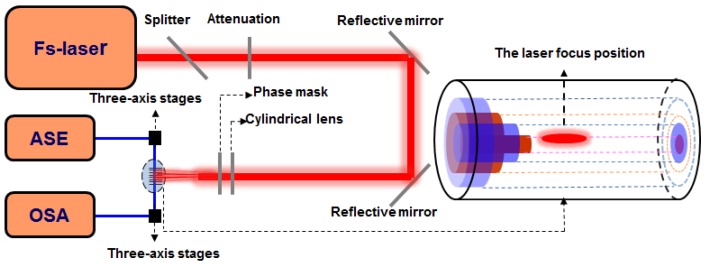
Schematic diagram of the experimental setup for “cladding” FBG fabrication.

**Figure 2:**
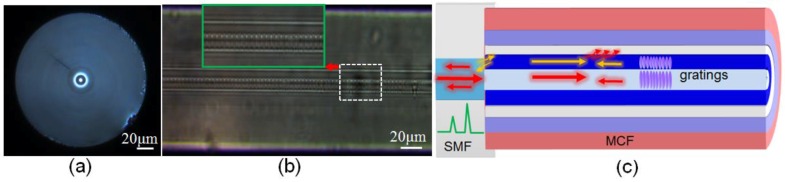
(**a**) Microscope image of the MCF cross-section. Inset shows the refractive index cross-section of the MCF. (**b**) Photomicrograph of the gratings; (**c**) Schematic diagram of mode-coupling inside fiber.

Figure 1 shows the schematic diagram of the FBG inscription system. The proposed grating is fabricated using a Ti:sapphire laser system. The laser outputs pulses of duration with a 1 kHz repetition rate, which emits a linearly polarized light with a central wavelength of approximately 800 nm. A section of 10 mm hydrogen-loaded (at 60 °C and a H_2_ pressure of 10 MPa for 15 days) multi-cladding fiber (produced by YOFC), with core and claddings of 5 μm and 14 μm, 20 μm, 36 μm, 120 μm, is self-aligned (no-offset) and spliced with a leading-in single mode fiber SMF using a commercial compact fusion splicer (Fujikura FSM-60S). The optical microscope image of quadruple cladding fiber cross-section is shown in Figure 2a. It is seen clearly that the MCF has a step refractive index (RI) profile via the RI difference of the dopant or material within the fiber. The core of the fiber is highly doped with germanium, which is surrounded by a deeply depressed-index cladding. Another cladding with higher RI wraps on the first-layer cladding, and two more depressed external claddings are next to it.

The laser beam is precisely focused along one side of the MCF core-cladding interface (~2 μm core offset) before inscription. The average pulse energy of the laser output is fixed at 0.65 mJ (controlled by an optical attenuator), which is optimized by trial and error. The exposure time lasts 60 s (i.e., 60,000 laser pulses) and then a 5 mm grating region in the fiber core and cladding can be achieved simultaneously. As in the zoomed photographic images shown in Figure 2b, the grating inscription region is located along one side of interface of the fiber core and inner cladding. The formation of the uniform periodic patterns is based on nonlinear light-material interactions involving nonlinear multiphoton absorption and ionization because of the high-intensity and ultrashort pulses, which is different from the UV-induced color-center photosensitivity [25,26]. In addition, those index changes are mediated by a densification from the nonlinear multiphoton ionization that causes local melting and rapid quenching in the dielectric material after the optical breakdown. Furthermore, the formation of cladding-FBG is consistent with type-II damage gratings [25,27].

**Figure 3:**
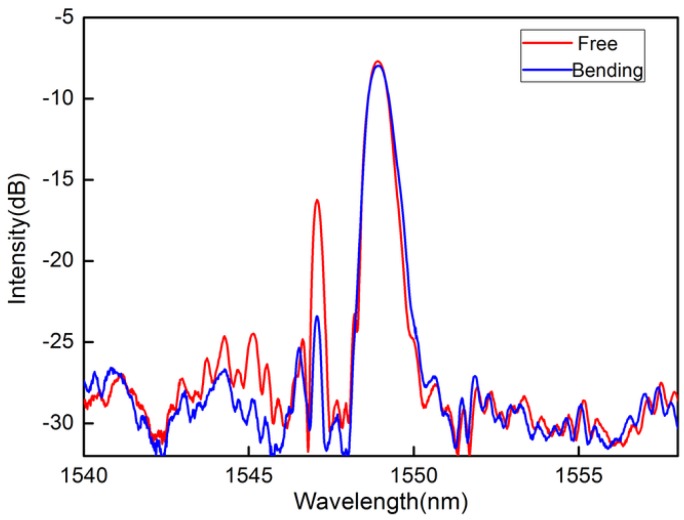
Spectra of QCF-FBG with and without bending.

Figure 2c shows the schematic diagram of the created grating structure configuration. The interface of the mismatch core between the SMF and QCF is used to forward the core-to-cladding mode coupling and the backward cladding-to-core recoupling. The cladding modes coupled and the core mode will get reflected by the downstream cladding and core FBG, the cladding mode resonance will partially be recoupled back to the upstream SMF, and eventually returns to the interrogation system. Therefore, two well-defined resonances in the reflections have been achieved. What is special is that the inner index-depressed cladding cannot confine the cladding modes well because of the special RI profile of the MCF. The fiber deformation not only influences the modes coupling at the splicing junction but also the propagation loss of the cladding modes in the inner cladding. Hence, the investigated sensor has a great response to fiber bending. In addition, because the effective refractive index (RI) difference between the core and inner cladding is 0.032, the resonant modes present a clear wavelength separation of 1.88 nm, and the center wavelengths of the reflection spectra are 1548.97 nm and 1547.09 nm, as shown by the red line in Figure 3.

In general, the RI of silica materials is modified by the change of the geometrical cross-section of the fiber which is caused by bending-induced anisotropic strain. Therefore, once this configuration is achieved, bending or deflecting the fiber introduces refractive index variations across the fiber that influence the reflection spectrum in several ways: the forward core-to-cladding mode coupling at the SMF-MCF splicing junction (forward coupling loss); the propagation loss of the cladding modes between the splicing junction and downstream FBG (bend loss); and the reflection loss of cladding modes between the first depressed-index cladding and second cladding because bending can introduce a strong first-to-second cladding coupling due to the downside of the depressed RI. Finally, the backward cladding-to-core recoupling at the SMF-MCF splicing junction will have a significant fluctuation. Among these effects, the change in the cladding-to-core mode recoupling at the splicing junction is thought to be dominant, especially in view of the behavior that the recoupled power decreases and increases around its unbent stage, as shown in Figure 3. In addition, the transverse intensity distribution of the mode in the MCF will be altered as fiber bending [28], caused by the RI change of the fiber. It will reduce the recoupling efficiency from the backward propagating cladding modes to the core of the upstream fiber. Therefore, the recoupled cladding mode will have an extremely high sensitivity to fiber bending. As a result, bending the fiber will induce a strong intensity modulation over the recoupled cladding mode but will have no effect on the core mode, and thus the power of the reflected core mode can be used as a reference to compensate for the unwanted power fluctuations. In addition, the cladding resonance presents high orientation dependence as the asymmetrical distribution of the cladding-FBG over the fiber cross-section.

## 3. Experiment Results and Discussion

**Figure 4:**
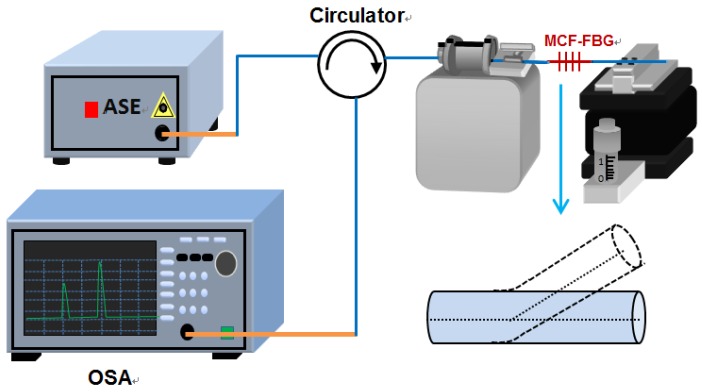
Schematic diagram of MCF-FBG as a displacement sensing system.

**Figure 5:**
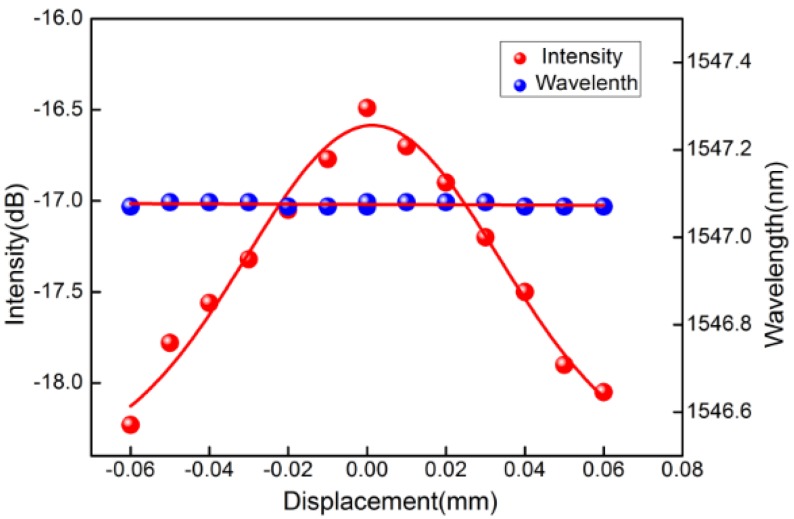
Cladding resonance mode power and wavelength versus displacements.

The schematic diagram for the displacement sensing system is shown in Figure 4. The light from an amplified spontaneous emission (ASE) is launched into the fabricated sensor through a circulator. The reflection light from the sensor is monitored by an optical spectrum analysis (OSA) with a wavelength resolution of 0.02 nm. In the experiment, one side of the sensing probe is held on a rotator at a fixed stage which can change the bending direction, and the other free end is fixed to a translation stage with a 10 μm resolution providing displacement along the vertical. The free-fiber length downstream is carefully selected to ensure that fiber bending can achieve the maximum effect on the cladding resonance mode power. The power of the reflected cladding mode decrease with the increasing fiber bending, while the resonance wavelength stays unchanged, as shown by the blue line in Figure 3. The device shows the response to bending with the highest sensitivity of 27.7 dB/mm at 60° ranging from −60 to +60 µm (like an inverted V-shape), as shown in Figure 5. Besides, both the intensity and the Bragg wavelength of the core mode remain unchanged.

**Figure 6:**
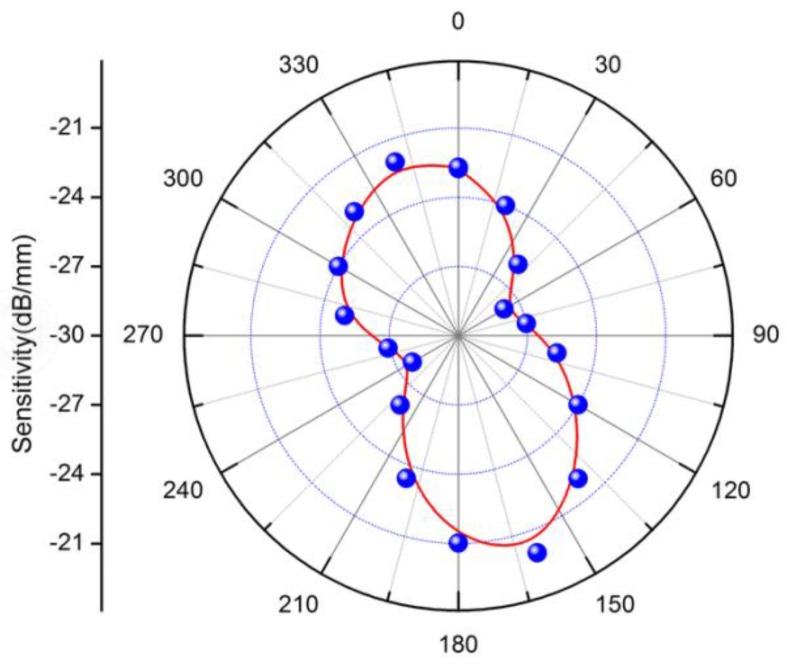
Angular dependence of the displacement responsivity of the sensor.

In order to characterize the bending orientation dependence of sensor, the fiber is rotated from 0° to 360° with a step of 20°, and the bending-induced intensity loss is recorded at each angle. The bending sensitivity of different orientations is calculated, and a strong angular dependence of the bending response has been achieved, as shown in Figure 6. It is caused by the asymmetrical distribution of the cladding grating over the fiber cross-section, which is similar to our previous work [23]. The maximum sensitivity is realized when the bending axis is parallel to the grating plates, and the minimum sensitivity results from the bending axis being at the orthogonal direction. It is important to note that the orientation function is not completely symmetrical. This behavior is mainly due to that the fact that the change of the effective RI for the cladding mode caused by different fiber bending orientations is different from the asymmetrical distribution of the cladding-FBG over the fiber cross-section.

**Figure 7:**
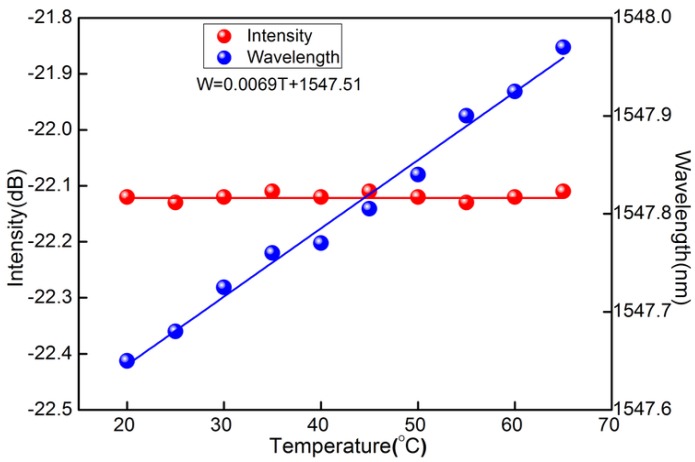
Temperature response performances of “cladding” FBG reflection resonance included wavelength and power fluctuation with increasing temperature.

The temperature response for the sensor is also investigated by placing the MCF-FBG in a heating oven with an accuracy of ±0.1 °C. The temperature is varied from 20 to 65 °C. For every temperature point, the temperature is kept constant for 20 min in order to ensure a well-distributed temperature around the sensor probe before each record. We plot the cladding resonance wavelength as the function of temperature, as shown in Figure 7. It is seen that the wavelength shift presents a linear sensitivity of 6.9 pm/°C whereas the intensity of the reflected cladding mode is almost kept unchanged with the temperature rising, as shown in Figure 7. Therefore, the displacement measurement is temperature independent, meanwhile giving it a potential for simultaneous measurements of displacement and temperature.

## 4. Conclusions 

In this paper, a novel FBG inscribed in a multi-cladding single-mode fiber over the core and depressed-index cladding by a femtosecond laser is proposed and experimentally demonstrated. A reflection spectrum with two defined resonant modes is obtained corresponding to the core mode and cladding mode. The FBG-based device is employed for displacement measurement, and its sensitivity shows high orientation dependence. Moreover, the construction can provide remote sensing as a reflection probe, and the fabrication is simple and effective, making it a good candidate for structural health monitoring.
